# NeMeHg, genetically encoded indicator for mercury ions based on mNeonGreen green fluorescent protein and merP protein from *Shigella flexneri*

**DOI:** 10.3389/fbioe.2024.1407874

**Published:** 2024-07-10

**Authors:** Oksana M. Subach, Kiryl D. Piatkevich, Fedor V. Subach

**Affiliations:** ^1^ Complex of NBICS Technologies, National Research Center, Kurchatov Institute, Moscow, Russia; ^2^ School of Life Sciences, Westlake University, Hangzhou, China; ^3^ Westlake Laboratory of Life Sciences and Biomedicine, Hangzhou, China; ^4^ Institute of Basic Medical Sciences, Westlake Institute for Advanced Study, Hangzhou, China

**Keywords:** biosensor, live cell imaging, mercury, fluorescent proteins, protein engineering

## Abstract

The detection of mercury ions is an important task in both environmental monitoring and cell biology research. However, existing genetically encoded sensors for mercury ions have certain limitations, such as negative fluorescence response, narrow dynamic range, or the need for cofactor supplementation. To address these limitations, we have developed novel sensors by fusing a circularly permutated version of the mNeonGreen green fluorescent protein with the merP mercury-binding protein from Gram-negative bacteria *Shigella flexneri*. The developed NeMeHg and iNeMeHg sensors responded to mercury ions with positive and negative fluorescence changes, respectively. We characterized their properties *in vitro*. Using the developed biosensors, we were able to successfully visualize changes in mercury ion concentration in mammalian cultured cells.

## Introduction

Heavy metals are hazardous substances that cause environmental pollution and pose health risks to humans and mammalians (Tchounwou et al., 2003; Tchounwou et al., 2012). Arsenic (As), cadmium (Cd), chromium (Cr), lead (Pb), and mercury (Hg) are particularly significant to human health due to their high toxicity. These heavy metals are considered systemic toxicants causing damage to multiple organs even at low exposure levels and are classified as human carcinogens. Consequently, the detection of heavy metals in the environment and within living cells is a critical challenge. Among numerous analytical and spectroscopic methods for heavy metal detection, genetically encoded sensors based on fluorescent proteins enable real-time visualization of heavy metal ions in various states: dissolved in solution, attached to cellular membranes, and within the cytoplasm of living cells (Burdette et al., 2001; Mills et al., 2013; Chen et al., 2023; Torres-Ocampo and Palmer, 2023).

Among the genetically encoded biosensors for heavy metal ions, those for zinc ions are the most numerous. For example, the BLCALWY-1 biosensor based on zinc-binding pair Atox1-WD4 (Aper et al., 2016), GZnP3 and RZnP1 sensors based on zinc finger domains Zap1 (Minckley et al., 2019; Dischler et al., 2022), FRISZ sensor based on minimal zinc finger pfRad50 (Wu et al., 2023). The luminescent BLCALWY-1 sensor has a large molecular size and its principle of operation is based on the change of BRET signal (maximum 12%) of luciferase NLuc as a result of the change of FRET signal in the Cerulean/Citrine pair upon binding of zinc ions by Atox1-WD4 proteins (Aper et al., 2016). The use of this type of sensors has great limitations due to their large size, low contrast (12%), and the expensive equipment required to detect BRET or FRET signals. The green or red fluorescence of GZnP3 and RZnP1 sensors based on circularly permutated cpEGFP and cpmApple changes with contrasts of 11 and 4, respectively, as a result of the binding of zinc ions by the zinc-binding factor Zap1, which consists of two zinc fingers ZP1 and ZP2 that interact to bind zinc ions (Minckley et al., 2019; Dischler et al., 2022). The fluorescence of the FRISZ zinc sensor increases 7-fold due to zinc-induced homodimerization of pfRad50 zinc fingers, causing a change in the far-red fluorescence of the circular-permutated protein cpmMaroon (Wu et al., 2023). Zinc sensor variants have different affinities for zinc ions, allowing detection of zinc ions in the cytosol of cells and outside the cell membrane; however, there is no contrast sensor with an affinity for zinc ions around 100 p.m. that is most optimal for zinc ion detection in the cytosol of mammalian cells. Thus, sensors for zinc ions are the most developed with high contrasts, small sizes and different affinities for zinc ions.

The second largest group of sensors includes copper ion sensors such as CreiLOV and miniGFPs based on light-oxygen-voltage (LOV) sensing domain (Zou et al., 2020; Liang et al., 2022), as well as Ace1-FRET, Mac1-FRET and Amt1-FRET based on copper-binding domains of Ace1 (36–100), Mac1 (203–295) and Amt1 (36–110) yeast transcriptional protein regulators (Wegner et al., 2010; Wegner et al., 2011). Sensors based on LOV domains are small in size and reduce fluorescence intensity in the presence of copper ions by 2–3 fold (Zou et al., 2020; Liang et al., 2022). Ace1-FRET, Mac1-FRET and Amt1-FRET sensors are large in size as they consist of two ECFP/EYFP fluorescent proteins and have low contrast in the order of 10%–15% (Wegner et al., 2010; Wegner et al., 2011). Thus, copper ion sensors have limited contrast.

The third group includes sensors for other heavy metal ions such as arsenic, cadmium, lanthanum, manganese and mercury. The arsenic sensor SenALiB changes the FRET signal by 8% between eCFP/mVenus proteins when arsenic ions (+3) are bound by the repressor protein ArsR from E.Coli (Soleja et al., 2019). SenALiB also responds to arsenic ions (+5). The Cd-FRET-2 sensor consisting of the FRET pair Citrine/Cerulean responds to cadmium ions with a contrast of 32% as a result of their binding by four cysteine residues in the dimerization interface of the two proteins (Vinkenborg et al., 2011). The cadmium sensor Cd-FRET-2 also responds to cobalt, nickel and lead ions with lower contrasts. The LaMP1 sensor for lanthanum ions (+3) consists of a FRET pair of ECFP/Citrine proteins and lanmodulin (LanM) from the bacteria Methylobacterium extroquens; upon lanthanum binding, LaMP1 changes the FRET signal 6-fold (Mattocks et al., 2019). However, the LaMP1 sensor for lanthanum reacts with similar contrasts to 15 other metal ions of the lanthanide group, and also reacts with about 2–3–fold lower contrasts to aluminum, manganese, and calcium ions. The sensor for manganese (2+) ions, MnLaMP2, consists of an ECFP/Citrine FRET pair and a modified version of the LanM protein that changes the FRET signal by a factor of 3-fold upon Mn^2+^ binding (Subach et al., 2012). The manganese sensor MnLaMP2 reacts further with magnesium and calcium ions with similar contrasts, but has a lower affinity for these ions. Two intensiometric sensors for mercury (2+) ions, eGFP205C and IFP, have only one fluorescent protein domain, which acts as both fluorescent and metal-binding domains (Chapleau et al., 2008; Gu et al., 2011). eGFP205C and IFP responded to the addition of mercury ions by quenching fluorescence 1.3- and 11.5-fold. However, IFP protein responded to mercury ions only upon simultaneous addition of the cofactor biliverdin (BV) or upon subsequent addition of BV. The IFP sensor itself did not react to mercury in the BV-bound state, which limits the application of IFP in mammalian cells since the latter contain BV. The eGFP205C and IFP with lower contrasts reacted to cobalt or copper ions, respectively. Thus, group III heavy metal sensors have only an inverse phenotype, limited contrast, low specificity, or require cofactor addition.

Many whole-cell mercury biosensors have been reported that utilize bacterial species carrying mercury-responsive regulators (MerR or ZntR) that produce different pigments or enzymes in the mercury dependent manner. Metalloregulators MerR and ZntR are a Hg(II)-responsive transcriptional factors, which has been employed to develop bacterial whole-cell biosensors using luciferase (Din et al., 2019), β-galactosidase or fluorescence proteins (Hansen and Sørensen, 2000; Kang et al., 2018; Zhang et al., 2021), and visual pigments violacein (Guo et al., 2021) and indigoidine (Hui et al., 2021) as the signal outputs.; These systems can detect mercury ions up to 6 nM. However, whole cell-based microbial sensors require viability and integrity of the cells, which substantially limits the variety, detection level and detection range of target metal ions (Lee and Kim, 2019) and they are not applicable for detection of the heavy ions in compartments of mammalian cells.

Thus, intensiometric sensors for heavy metal zinc ions based on a single fluorescent protein are the most developed in terms of minimal molecule size, high contrast, and varying affinity for zinc ions. However, single-FP-based sensors for other heavy metals are either not available or less developed. Thus, there is a need for sensors for heavy metal ions based on a single fluorescent protein with high contrast.

In this work, we first developed sensors based on a single circularly permutated fluorescent protein mNeonGreen and MerP protein from *Shigella flexineri*. The NeMeHg and iNeMeHg sensor respond to the addition of mercury (2+) ions by increasing and decreasing fluorescence with contrasts ΔF/F of 2.2 and −2.02, respectively. We characterized the molecular brightness and spectral properties of the developed NeMeHg and iNeMeHg sensors in solution. We characterized the affinity and specificity of the developed sensors towards mercury ions *in vitro*. Using NeMeHg and iNeMeHg sensors, we were able to successfully detect changes in the concentration of mercury ions in the cytosol of mammalian cells.

## Results

### Development of NeMeHg and iNeMeHg sensors in a bacterial system

**FIGURE 1:**
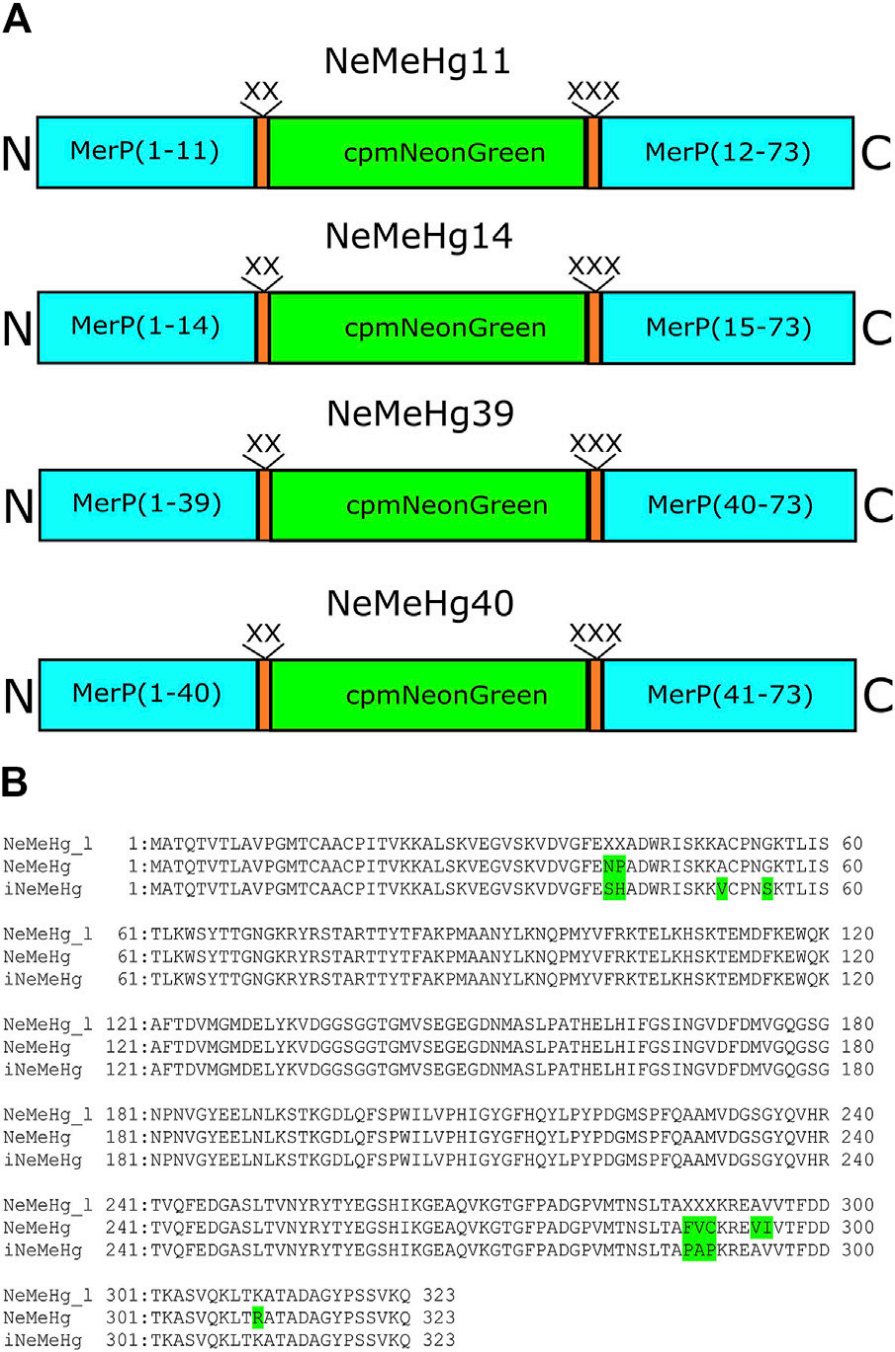
Structure of the initial sensor libraries and amino acid sequence alignment of the initial library with randomized NeMeHg_l (NeMeHg40) linkers and NeMeHg and iNeMeHg sensors. **(A)** MerP, linkers, and cpmNeonGreen are shown in cyan, orange, and green, respectively. **(B)** Mutations relative to the original library are highlighted in green.

To develop the NeMeHg and iNeMeHg sensors, site-directed and random bacterial libraries were generated, expressed, and subsequently analyzed on crude bacterial extracts. The merP protein gene from *Shigella flexneri*, a periplasmic protein that binds mercury ions and transfers them to the membrane transport protein merT, was synthesized *de novo* from primers (Table 1). The merP protein was chosen as the mercury-binding domain because X-ray structures in mercury-bound and free states are available for it (Steele and Opella, 1997). mNeonGreen was chosen as the fluorescent domain because the NCaMP7 (Subach et al., 2020) and mNG-GECO1 (Zarowny et al., 2020) calcium indicators derived from mNeonGreen have the highest molecular brightness among the available calcium indicators. A circularly permutated version of the mNeonGreen protein (cpmNeonGreen) was taken from a variant of the calcium sensor NCaMP19-11 obtained previously in our laboratory (unpublished data); Four insertion sites of the cpmNeonGreen protein into the merP protein were chosen according to analysis of the structures of the merP protein in the ligand-bound and free states; at these sites, according to the structure, maximum conformational changes occur upon mercury ion binding. Four rational libraries were obtained in which cpmNeonGreen was inserted after residues 11, 14, 39, and 40 in the merP protein. The linkers (L1 = XX, L2 = XXX) between merP and cpmNeonGreen proteins were randomized and corresponding rational libraries were screened (Figure 1A). For each library, the brightest colonies were selected from about 20,000 colonies on Petri dishes (2–3 colonies per dish). The colonies on Petri dishes were grown at 37°C for 24 h, and then incubated at room temperature for 24 h to ensure complete folding and maturation of the sensors. The response of the selected mutants to the addition of mercury ions (2+) was further tested on bacterial lysates in a 96-well format on a platereader. As a result, clones with contrasts of 0.84, −0.2, and 0.75, 0.74, −0.39, and 0.73 were found in the NeMeHg11, NeMeHg14, NeMeHg39, and NeMeHg40 libraries, respectively.

**TABLE 1:** List of primers.

Primer	Sequence 5′-3′
BglII-MerP	gac​AGA​TCT​ATG​GCT​ACC​CAG​ACC​GTC
MerP-1	ATG​GCT​ACC​CAG​ACC​GTC​ACG​CTA​GCG​GTT​CCC​GGC​ATG​ACT​TGC​GCC​GCC​TGC​CCG​ATC​ACA​GTC​AAG
MerP-2-r	CTC​GAA​GCC​CAC​ATC​GAC​CTT​GCT​CAC​GCC​TTC​GAC​CTT​GGA​GAG​CGC​TTT​CTT​GAC​TGT​GAT​CGG​GCA​G
MerP-3	GTC​GAT​GTG​GGC​TTC​GAG​AAG​CGC​GAG​GCC​GTC​GTC​ACT​TTT​GAC​GAC​ACC​AAG​GCC​AGC​GTA​CAG​AAG​C
MerP-4-r	TCA​CTG​CTT​GAC​GCT​GGA​CGG​ATA​GCC​GGC​GTC​TGC​GGT​GGC​CTT​GGT​CAG​CTT​CTG​TAC​GCT​GGC​CTT​G
MerP-EcoRI-r	tcg​aat​tcT​CAC​TGC​TTG​ACG​CTG​GAC​GG
Mer11	ATGGCTACCCAGACCGTCACGCTAGCGGTTCCCGGCNNTNNTGCTGACTGGCGTATATCCAAG; 0.04
N-Mer11	GACAAATTCCCTGACAGCANNTNNTNNTATGACTTGCGCCGCCTGC
N-Mer11-r	GCAGGCGGCGCAAGTCATANNANNANNTGCTGTCAGGGAATTTGTC
BglII-Mer14	ATGGCTACCCAGACCGTCACGCTAGCGGTTCCCGGCATGACTNNTNNTGCTGACTGGCGTATATCCAAG
N-Mer14	GACAAATTCCCTGACAGCANNTNNTNNTTGCGCCGCCTGCCCGATC
N-Mer14-r	GATCGGGCAGGCGGCGCAANNANNANNTGCTGTCAGGGAATTTGTC
Mer39-N	CAAGGTCGATGTGGGCTTCNNTNNTGCTGACTGGCGTATATCCAAG
Mer39-N-r	CTTGGATATACGCCAGTCAGCANNANNGAAGCCCACATCGACCTTG
N-Mer39	GACAAATTCCCTGACAGCANNTNNTNNTGAGAAGCGCGAGGCCGTCG
N-Mer39-r	CGACGGCCTCGCGCTTCTCANNANNANNTGCTGTCAGGGAATTTGTC
Mer40-N	GGTCGATGTGGGCTTCGAGNNTNNTGCTGACTGGCGTATATCCAAG
Mer40-N-r	CTTGGATATACGCCAGTCAGCANNANNCTCGAAGCCCACATCGACC
N-Mer40	GACAAATTCCCTGACAGCANNTNNTNNTAAGCGCGAGGCCGTCGTC
N-Mer40-r	GACGACGGCCTCGCGCTTANNANNANNTGCTGTCAGGGAATTTGTC

The clones in the NeMeHg40 library were 2–3 times brighter, so these clones were chosen as templates for several rounds of random mutagenesis. Random libraries were screened as described above for rational libraries. After 3 rounds of random mutagenesis, final versions named NeMeHg and iNeMeHg (NeonGreen and MerP-based sensor for Hg^2+^) with positive and inverted responses to mercury ion addition were obtained, respectively. According to the alignment of the amino acid sequences of NeMeHg and iNeMeHg with the original rational library, in addition to five linker mutations, the sensors contained three and two additional mutations (Figure 1B).

### Characterization of the properties of NeMeHg and iNeMeHg sensors *in vitro*

**FIGURE 2:**
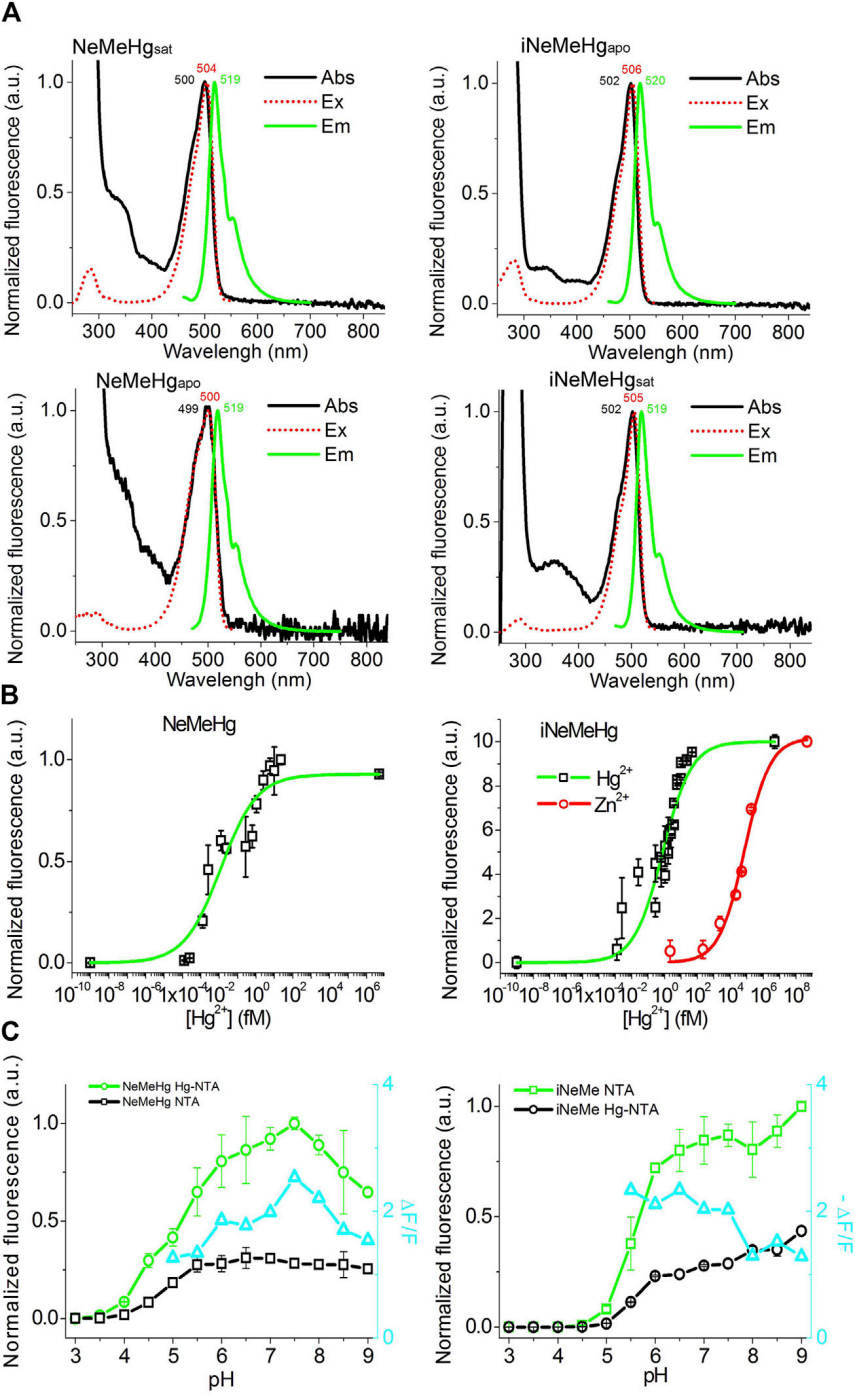
Physicochemical and spectral properties of NeMeHg and iNeMeHg sensors. **(A)** Absorption, excitation and fluorescence spectra in buffer **(B)** 50 mM Tris-HCl, 100 mM NaCl, 1 mM MgCl_2_, 10 mM HgNTA (NeMeHgsat and iNeMeHgsat) and in buffer **(A)** 50 mM Tris-HCl, 100 mM NaCl, 1 mM MgCl_2_, 10 mM NTA (NeMeHgapo and iNeMeHgapo). **(B)** Equilibrium binding curves of mercury or zinc ions by NeMeHg and iNeMeHg sensors. Buffers **(A,B)** were mixed in different ratios to vary the concentration of free mercury ions in the range of 0–5 nM. Buffers **(A,C)** (50 mM Tris-HCl, 100 mM NaCl, 1 mM MgCl_2_, 10 mM HgNTA) were mixed in different ratios to vary the concentration of free zinc ions in the range of 0–468 nM. **(C)** pH titration of the NeMeHg and iNeMeHg indicators in Hg^2+^-bound and Hg^2+^-free states.

We then characterized the spectral and physicochemical properties of NeMeHg and iNeMeHg purified from bacteria. The NeMeHg sensor in the mercury-bound state and iNeMeHg sensor in the apo-state exhibited absorption/excitation/emission maxima at 500/504/519 and 502/506/520 nm, respectively (Figure 2A; Table 2). The NeMeHg sensor in the apo-state and iNeMeHg sensor in the apo-state exhibited absorption/excitation/emission maxima at 499/500/519 and 502/505/519 nm, respectively (Figure 2A; Table 2). The molecular brightness, defined as the product of the extinction coefficient (determined by the alkaline denaturation method) by the quantum yield, was 36% and 64% of that of the meGFP protein or 2.8- and 1.6-fold lower than that of the standard meGFP protein (Table 2).

**TABLE 2:** Properties of NeMeHg and iNeMeHg sensors isolated from bacteria.

		NeMeHg_sat_	iNeMeHg_apo_
Maxima abs./exc./emis., nm		500/504/519 (499/500/519)^a^	502/506/520 (502/505/519)^b^
Exc. coeff., mМ^−1^cm^-1^ ^c^		40 ± 2	57 ± 3
Quantum yield^d^		0.35 ± 0.02	0.44 ± 0.02
Brightness vs. meGFP, %^e^		36	64
ΔF/F		2.2 ± 0.1	−2.02 ± 0.06
К_d_, fM^f^	Hg^2+^	0.012 ± 0.006 [0.44 ± 0.08]	0.76 ± 0.19 [0.50 ± 0.08]
	Zn^2+^	ND	74,000 ± 21,000 [0.56 ± 0.09]
pK_a_		5.18 ± 0.10 (4.92 ± 0.04)^a^	5.66 ± 0.10 (5.94 ± 0.01)^b^

^a^ Value in the brackets corresponds to the apo-state.

^b^ Value in the brackets is shown for saturated-state. ND, not determined.

^c^ Absorption coefficients were determined in 1 M NaOH, solution, assuming the absorbance of the GFP, chromophore under these conditions to be 44,000 M−1cm−1.

^d^ Quantum yield was determined at an excitation light wavelength of 470 nm, relative to meGFP, with a quantum yield of 0.70.

^e^ Brightness was determined as the product of quantum yield by extinction coefficient normalized to eGFP, brightness equal to 100%, assuming that the quantum yield and extinction coefficient for eGFP, are 0.70 and 56 mM−1cm−1.

^f^ Hill coefficient is indicated in square brackets.

Titration of NeMeHg and iNeMeHg sensors with mercury ions showed an affinity for mercury ions of 0.012 ± 0.006 and 0.76 ± 0.19 fM, respectively (Figure 2B). According to the Hill coefficient values for NeMeHg and iNeMeHg of 0.44 ± 0.08 and 0.50 ± 0.08, respectively, the sensors do not cooperatively bind mercury ions (Table 2). The maximum achievable ΔF/F contrasts for mercury ion binding by NeMeHg and iNeMeHg sensors reached values of 2.2 ± 0.1 and −2.02 ± 0.06, respectively (Table 2). It is known that mercury ion binding is sensitive to the redox state of the thiol group of the cysteine residues in the N-terminal part of merP. After the storage of the NeMeHg and iNeMeHg sensors for 24 h at room temperature in the absence of the reducing agent TCEP, their ΔF/F contrasts dropped by 1.17-fold or increased by 34-fold, respectively. The change in the ΔF/F contrasts was due to the decrease in the fluorescence of the Hg^2+^-bound state.

**FIGURE 3:**
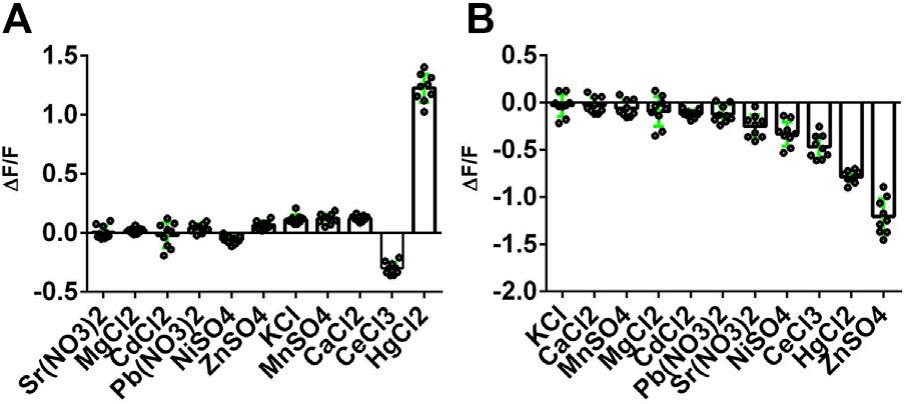
Selectivity of NeMeHg and iNeMeHg sensors to different metal ions. The fluorescence of proteins incubated at room temperature in 50 mM Tris-HCl, 100 mM NaCl, 100 mM NaCl, 1 mM MgCl_2_, 10 mM Mt-NTA buffer (where Mt is Sr, Mg, Cd, Pb, Ni, Zn, K, Mn, Ca, Ce, or Hg) was compared with the fluorescence of proteins in 50 mM Tris-HCl, 100 mM NaCl, 1 mM MgCl_2_, 10 mM NTA buffer. The part labels correspond to the fluorescence contrast values for the depicted metal ions.

We then tested the specificity of the response of the NeMeHg and iNeMeHg sensors to mercury ions by testing the response of the sensors to the addition of other metal ions. The NeMeHg sensor showed the maximum positive fluorescence response only to the addition of mercury ions (Figure 3A). The positive response to calcium, manganese and potassium ions was 10–11 times less (Figure 3A). The NeMeHg sensor showed virtually no response to the addition of other metal cations (ΔF/F response was 20-fold or smaller than the ΔF/F response to mercury ions), except for cerium ions, to which the sensor responded by quenching fluorescence but with a 4-fold smaller ΔF/F contrast than the contrast to mercury ions (Figure 3A). The inverted iNeMeHg sensor responded to the addition of zinc ions with 1.5-fold higher contrast ΔF/F (Figure 3B). However, titration of the iNeMeHg sensor with zinc ions showed that the sensor had a 97,000-fold lower affinity for zinc ions compared to mercury ions (Figure 2B; Table 2). The iNeMeHg also showed a response to cerium, nickel, strontium, cadmium, and magnesium ions with 1.7–8.5 times lower contrast than the contrast to mercury ions (Figure 3B). The fluorescence response of ΔF/F to other metal ions was negligible (13- to 26-fold less than to mercury ions; Figure 3B).

Next, we tested the pH sensitivity of the sensors, which would provide valuable information for application in intracellular environments. In the Hg^2+^-saturated and Hg^2+^-free states the NeMeHg and iNeMeHg indicators had pK_a_ values of 5.18 and 5.66, respectively (Figure 2C; Table 1). In the Hg^2+^-free and Hg^2+^-saturated states the NeMeHg and iNeMeHg indicators had pKa values of 4.92 and 5.94, respectively (Figure 2C; Table 1). Different pKa values in Hg^2+^-bound and Hg^2+^-free states resulted in the dependence of the ΔF/F response of the sensors on pH (Figure 2C). Calcium indicators based on a single GFP domain such as GCaMPs and NCaMP7 had similar pH sensitivity to pH (Chen et al., 2013; Subach et al., 2020). Hence, both NeMeHg and iNeMeHg were sensitive to the pH changes, similar to other types of GFP-based sensors.

Thus, we have characterized the spectral properties of the NeMeHg and iNeMeHg sensors, their molecular brightness, the affinity and dynamic range of the sensors to mercury ions, their selectivity toward mercury ions and pH stability.

### Characterization of the properties of NeMeHg and iNeMeHg sensors in mammalian cells

**FIGURE 4:**
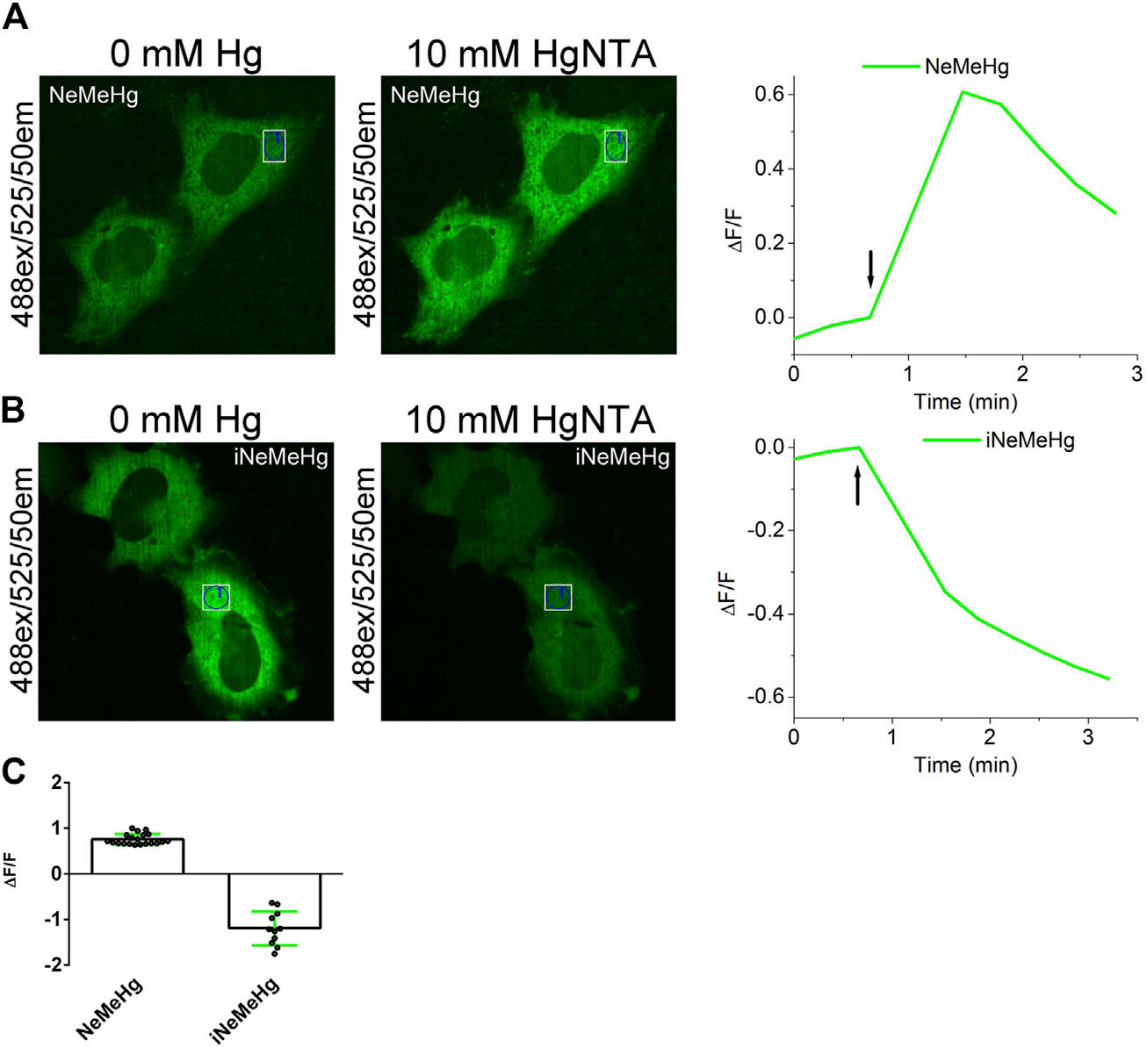
Visualization of mercury ion concentration changes in HeLa cells using NeMeHg and iNeMeHg sensors and confocal fluorescence microscopy. (a,b, left and middle panels) Confocal images of HeLa cells expressing NES-NeMeHg **(A)** or NES-iNeMeHg **(B)** before and after addition of 2.5 µM ionomycin and 10 mM HgNTA, 5 mM Tris-HCl, pH 7.40. [**(A,B)** right panel] Time dependence of the change in ΔF/F green fluorescence of the NeMeHg **(A)** or iNeMeHg **(B)** sensors in the indicated region of the cell cytosol in the left and middle panels before and after addition of 10 mM Hg-NTA and 2.5 µM ionomycin at the time indicated by the arrow. **(C)** Averaged ΔF/F responses of NeMeHg and iNeMeHg sensors in the cytosol of HeLa cells to the addition of 10 mM Hg-NTA and 2.5 μM ionomycin.

To characterize the properties of NeMeHg and iNeMeHg sensors in mammalian cells, they were transiently expressed in the cytosol of cells and changes in mercury ion concentration were visualized using confocal fluorescence microscopy. Human HeLa cancer cells were transiently transfected with plasmids pAAV-CAG-NES-NeMeHg and pAAV-CAG-NES-iNeMeHg (NES - nuclei-exclusion signal). 48–72 h after transfection, green fluorescence of NeMeHg and iNeMeHg sensors was observed, uniformly, distributed in the cytosol of the cells (Figures 4A, B). After addition of 2.5 µM ionomycin and 10 mM HgNTA (corresponding to 16 nM concentration of free mercury ions), we observed an increase or decrease in green fluorescence of NeMeHg and iNeMeHg sensors (Figures 4A, B) with averaged ΔF/F responses equal to 0.75 ± 0.12 and −1.2 ± 0.4, respectively (Figure 4C). Thus, NeMeHg and iNeMeHg sensors allow visualization of changes in mercury ion concentration in the cytosol of mammalian cells.

While iNeMeHg’s affinity for zinc ions is 97,000-fold lower than its affinity for mercury ions, the endogenous concentration of zinc ions is expected to be much higher than mercury in the cytosol of the cells (about 100 p.m.). The K_d_ affinity of the iNeMeHg sensor of 74 p.m. (Table 2) is close to the concentration of the zinc ions in the cytosol of the mammalian cells, and this might interfere with intracellular mercury ions transient measurements using iNeMeHg. This prompted us to test the sensitivity of the iNeMeHg indicator transiently expressed in the cytosol of the mammalian cells to the external addition of the zinc ions. After the addition of 2.5 µM ionomycin and 1 mM ZnNTA (corresponding to 148 nM concentration of free zinc ions), we practically did not observe changes in green fluorescence of the iNeMeHg sensor with averaged ΔF/F response equal to 0.061 ± 0.007 (two cultures, 6 cells). Hence, the iNeMeHg sensor practically did not respond to zinc ion transients in the cytosol of mammalian cells.

## Discussion

Here, we report engineering NeMeHg and iNeMeHg biosensors based on a single fluorescent protein mNeonGreen, developed by a directed molecular evolution approach in a bacterial system; the NeMeHg and iNeMeHg sensors responded with high specificity to mercury ions by increasing or decreasing green fluorescence by a ΔF/F factor of 2.2 ± 0.1 and −2.02 ± 0.06, respectively. Both sensors were characterized *in vitro* and were able to detect changes in mercury ion concentration in the cytosol of mammalian cells.

Compared to the published mercury ion sensors eGFP205C and IFP, the NeMeHg and iNeMeHg sensors have several advantages. The NeMeHg sensor, unlike the eGFP205C and IFP sensors, has a positive response to mercury ions, making it more convenient to visualize mercury ions. Compared to eGFP205C sensor, NeMeHg and iNeMeHg sensors show 6-fold larger contrast to mercury ions, and have 10^8^–10^10^-fold higher affinity for mercury ions (Chapleau et al., 2008). Unlike the NeMeHg and iNeMeHg sensors, the molecular brightness of eGFP205C has not been determined and its application in mammalian cells has not been shown. The IFP sensor has 2- and 4-fold lower molecular brightness compared to NeMeHg and iNeMeHg sensors, respectively (Gu et al., 2011).

The IFP sensor has a 10^9^–10^19^-fold lower affinity for mercury ions than the NeMeHg and iNeMeHg sensors. The IFP sensor responds to mercury ions only when the cofactor biliverdin and mercury ions are added simultaneously, making it difficult to use both *in vitro* and in mammalian cells. Since mammalian cells have endogenous biliverdin, most of the IFP protein will already be bound to it and will not be able to respond to the addition of mercury ions, which has been shown for mammalian HEK-293 cells to result in a 4-fold decrease in contrast compared to *in vitro* contrast. Moreover, the IFP sensor contrast will have different values in different cells with different levels of endogenous biliverdin. The NeMeHg and iNeMeHg sensors are devoid of these limitations.

The ΔF/F responses of the iNeMeHg and NeMeHg sensors to the addition of the Hg^2+^ ions in the cytosol of the mammalian cells (Figure 4C) were 1.7- and 2.9-fold lower as compared to the responses of the respective sensor in a buffer (Table 2). The observed differences correlated with the sensors’ different affinity. Hence, the affinities of the sensors were not optimal for the registration of the mercury levels in the cytosol of mammalian cells, and further optimization of the sensors’ affinity to the mercury ions would be beneficial.

## Conclusion

We developed the NeMeHg and iNeMeHg sensors for mercury ions, which are currently the best sensors for mercury ions with high contrast, high molecular brightness, high specificity and affinity for mercury ions, and NeMeHg and iNeMeHg do not require the addition of cofactors.

## Experimental procedures

### Gene synthesis, cloning, and library generation

The merP gene was synthesized by polymerase chain reaction (PCR) with overlapping primers (Table 1). The gene was cloned at the BglII/EcoRI restriction sites into the pBAD/HisB plasmid and transfected into March1 cells.

Libraries with randomized linkers were obtained by overlapping fragment PCR (Ho et al., 1989) using primers (Table 1). Libraries were cloned by BglII/EcoRI restriction sites into pBAD/HisB plasmid and transfected into BW25113 cells.

Random libraries were obtained by PCR in the presence of manganese ions under conditions of 2–3 mutations per 1,000 base pairs. The libraries were cloned by BglII/EcoRI restriction sites into pBAD/HisB plasmid and transformed into BW25113 cells.

### Libraries screening

Bacterial libraries were first analyzed on Petri dishes under a Leica fluorescence microscope. Green fluorescence was recorded using 480/40 nm and 535/40 nm filters. The brightest colonies were selected.

The selected variants were then analyzed on lysates. For this purpose, selected clones were inoculated with 5 mL of LB medium containing ampicillin (100 μg/mL) and protein expression inducer arabinose (0.004%) and grown overnight at 37°С, 220 rpm. Cells were precipitated at 3,500 rpm, 12 min, the precipitate was frozen and 150 μL of BPer extraction reagent containing lysozyme (1 μg/mL) and benzonase (1.25 units/mL) was added. Incubation was performed at 37°С, 20 min, 220 rpm. The cell lysate was clarified by centrifugation at 20,000 *g*, 2 min. Then, 200 μL of buffer A (50 mM Tris-HCl, 100 mM NaCl, 100 mM NaCl, 1 mM MgCl_2_, 10 mM NTA) and 200 μL of buffer B (50 mM Tris-HCl, 100 mM NaCl, 1 mM MgCl_2_, 10 mM Hg-NTA) and recorded green fluorescence (488/12 nm and 535/12 nm) on a plate reader (background fluorescence). Then, 10 μL of lysate was lysed into a 96-well plate in 200 μL of buffer A and 200 μL of buffer B and green fluorescence (488/12 nm and 535/12 nm) was recorded on a flatbed reader. The values of ΔF/F were calculated according to the formulas: ΔF/F=(I_Hg-NTA_-I_background1_)/(I_NTA_-I_background2_)-1 and ΔF/F = −[(I_NTA_-I_background1_)/(I_Hg-NTA_-I_background2_)-1] for positive and inverted phenotypes, respectively, where I_Hg-NTA,_ I_NTA,_ I_background_ are fluorescence intensities for Hg^2+^-bound-state, apo-state, and background, respectively. For the top selected variants, the measurement of the F/F values was repeated three times.

### Protein purification and characterization *in vitro*

To characterize the spectral properties of purified proteins, the proteins contained HisB- or HisB-SUMO-tag at their N-terminus were isolated from 250 mL of LB medium containing ampicillin (100 μg/mL) and protein expression inducer arabinose (0.004%) and grown overnight at 37^o^С, 220 rpm. Cells were precipitated at 5000 g for 10 min. The precipitate was then resuspended in 10 mL of 30 mM MOPS, 0.5 mM TCEP, 10 mM imidazole, pH 7.20 and cells were disrupted by sonication for 4 min at 20% power. The cell lysate was clarified by centrifugation at 18000 *g*, 10 min. Protein was then bound to 500 µL of Ni-NTA resin (1:1 suspension) for 1 h at 4^o^С. After washing the resin 3 times with 5 mL of 30 mM MOPS, 0.5 mM TCEP, 10 mM imidazole, pH 7.20, protein elution was performed in 400 μL in 400 mM imidazole, 30 mM MOPS, 0.5 mM TCEP, pH 7.20 buffer. Proteins were dialyzed at 4^o^С 24 h opposite 1 L of 30 mM MOPS, 0.5 mM TCEP, 100 μM NTA, pH 7.20 buffer and another 24 h at 4^o^С opposite 1 L of 30 mM MOPS, 0.5 mM TCEP, pH 7.20 buffer.

To determine the values of dissociation constants (K_d_), green fluorescence of the sensors (0.5 μM final concentration) was recorded after incubation at room temperature for 20–30 min in buffers A: 50 mM Tris-HCl, pH 7. 20, 100 mM NaCl, 100 mM NaCl, 1 mM MgCl_2_, 10 mM NTA and buffer B: 50 mM Tris-HCl, pH 7. 20, 100 mM NaCl, 1 mM MgCl_2_, 10 mM Hg-NTA mixed in ratios of 10:0, 19,999:1, 9999:1, 1999:1, 999:1, 199:1, 199:1, 99:1, 9:1, 8:2, 7:3, 6:4, 5:5, 4:6, 3:7, 2:8, 1:9, 1:9.5, and 0:10. Free mercury ion concentration was calculated using the formula: [Hg^2+^]_free_ = K_d_*[HgNTA]/[NTA], Γде K_d_ (HgNTA) = 2.51*10^−15^ М. For a ratio of [HgNTA]: [NTA] = 10:0, a different equation was used: ${\left[{Hg}^{2+}\right]}_{free}=\sqrt{K_{d}*\left[HgNTA\right]}$ , where K_d_ (HgNTA) = 2.51*10^−15^ М. Kd values and Hill coefficients for mercury ion binding by sensors were calculated by nonlinear regression of experimental points by the Hill equation: $I=I_{\max}\frac{{\left[{Hg}^{2+}\right]}^{n}}{K_{d}^{n}+{\left[{Hg}^{2+}\right]}^{n}}$ , where I is the fluorescence intensity at a certain concentration of mercury, and I_max_ is the fluorescence intensity at the plateau at saturating concentrations of mercury ions. Titration with mercury ions was performed similarly to mercury ions by mixing buffer A with buffer C: 50 mM Tris-HCl, pH 7.20, 100 mM NaCl, 1 mM MgCl_2_, 10 mM Hg-NTA.

To test the selectivity of NeMeHg and iNeMeHg sensors to different metal ions, fluorescence of purified proteins (0. 5 μM final concentration) incubated at room temperature for 5–10 min in buffer 50 mM Tris-HCl, 100 mM NaCl, 1 mM MgCl_2_, 10 mM Mt-NTA (where Mt is Sr, Mg, Cd, Pb, Ni, Zn, K, Mn, Ca, Ce, or Hg) was compared with the fluorescence of proteins in 50 mM Tris-HCl, 100 mM NaCl, 1 mM MgCl_2_, 10 mM NTA buffer. Green fluorescence (488/12 nm and 535/12 nm) was recorded on a plate reader. The ΔF/F values were calculated according to the formulas: ΔF/F=(I_Hg-NTA_-I_background1_)/(I_NTA_-I_background2_)-1 and ΔF/F = −[(I_NTA_-I_backgroundNTA_)/(I_Hg-NTA_-I_backgroundHg-NTA_)-1] for positive and inverted phenotypes, respectively, where I_Hg-NTA,_ I_NTA,_ I_background_ are fluorescence intensities for Hg^2+^-bound-state, apo-state, and background, respectively.

For all experiments we used proteins containing a HisB-SUMO-tag at their N-terminus, except for the metal ions selectivity experiment, in which proteins contained a HisB-tag at their N-terminus.

### Statistical processing of the results

Figures represent mean values ± standard error throughout.

## Data Availability

Source files for the figures are available at FigShare (https://doi.org/10.6084/m9.figshare.26003779). The sequences for NeMeHg and iNeMeHg are deposited in the GenBank databases (accession numbers PP954959 and PP954960, respectively). Full-length sequences of the generated plasmids are available from WeKwikGene (https://wekwikgene.wllsb.edu.cn/) with the following accession codes: pBAD-HisB-Sumo-NeMeHg #0 0000571; pAAV-AscI-CAG-NES-NeMeHg #0000572; pBAD-HisB-Sumo-iNeMeHg #0000573; pAAV-AscI-CAG-NES-iNeMeHg #0000574.
